# GLX351322, a Novel NADPH Oxidase 4 Inhibitor, Attenuates TMJ Osteoarthritis by Inhibiting the ROS/MAPK/NF-*κ*B Signaling Pathways

**DOI:** 10.1155/2023/1952348

**Published:** 2023-01-30

**Authors:** Jinze Zhen, Xinwei Chen, Yi Mao, Xinru Xie, Xuzhuo Chen, Weifeng Xu, Shanyong Zhang

**Affiliations:** Department of Oral Surgery, Shanghai Key Laboratory of Stomatology & Shanghai Research Institute of Stomatology, National Clinical Research Center for Oral Diseases, Shanghai Ninth People's Hospital, College of Stomatology, Shanghai Jiao Tong University School of Medicine, Shanghai 200011, China

## Abstract

As a degenerative disease in joints, temporomandibular joint osteoarthritis (TMJOA) is characterized by progressive cartilage degradation, subchondral bone remodeling, and chronic synovitis, severely undermining functions and quality of life in patients. NADPH oxidase 4 (NOX4) contributes to reactive oxygen species (ROS) production and inflammatory pathway activation in osteoarthritis, which has attracted increasing attention in research in recent years. GLX351322 (GLX), a novel NOX4 inhibitor, exerts a protective effect on chondrocytes. However, whether it has a therapeutic effect on ROS production and inflammatory responses in synovial macrophages remains to be evaluated. In this study, we examined the effect of GLX on LPS-induced ROS production and inflammatory responses *in vitro* and on complete Freund's adjuvant (CFA)-induced TMJ inflammation *in vivo*. We found that GLX could depress LPS-induced intracellular ROS production and inflammatory response without cytotoxicity by inhibiting the ROS/MAPK/NF-*κ*B signaling pathways. In line with *in vitro* observations, GLX markedly attenuated the synovial inflammatory reaction in the TMJ, thus protecting the condylar structure from severe damage. Taken together, our results suggest that GLX intervention or NOX4 inhibition is a promising curative strategy for TMJOA and other inflammatory diseases.

## 1. Introduction

Temporomandibular joint osteoarthritis (TMJOA) is a degenerative joint disease characterized by progressive cartilage degeneration, subchondral bone remodeling, and chronic synovitis [1, 2]. As a common TMJ disorder, TMJOA is often secondary to anterior disc displacement, trauma, excessive joint loading, and dental-craniofacial deformities, resulting in severe joint dysfunction [2]. In recent years, epidemiological studies have found that the incidence of TMJOA increases with age and affected patients tend to be younger [3]. The clinical manifestations of TMJOA include pain, abnormal sounds, restricted mouth opening, jaw movement disorders, and dentofacial deformities, which seriously undermine the chewing, articulation, and even psychology of patients [2]. Although the current treatment modalities, including conservative treatment and surgical intervention, help alleviate symptoms and restore joint function, long-term stability remains unsatisfactory, with numerous adverse side effects [4, 5]. Therefore, it is imperative to explore new curative strategies for TMJOA management.

However, the pathogenesis of TMJOA remains unclear. Articular cartilage degeneration caused by abnormal mechanical loading is a well-recognized initiating factor of OA and has been the focus of research in this field [6, 7]. However, increasing evidence has indicated an important role of reactive synovial inflammation in the progression of TMJOA [8, 9]. Synovitis is highly associated with some clinical symptoms and signs of OA, such as pain, joint swelling, and effusion, which can greatly reflect disease progression [10–12]. Increased number and infiltration of macrophages in the synovial lining are the main pathological features of synovitis, histologically characterized by thickening of synovial tissue [13]. During TMJOA progression, synovial macrophages can be activated in various ways, the most important one of which involves the activation of pattern recognition receptors (PRRs) on the surface of macrophages, which identify endogenous danger-associated molecular patterns (DAMPs) and exogenous pathogen-associated molecular patterns (PAMPs). Activated PRRs can initiate intracellular downstream signaling pathways, including the PI3K/AKT, mitogen-activated protein kinase (MAPK), and nuclear factor kappa-B (NF-*κ*B) signaling pathways, thereby increasing the secretion of inflammatory cytokines [14, 15]. In an OA environment, cartilage detritus, disc debris, proteoglycans, fibronectin, and necrotic cells represent DAMPs that stimulate macrophage activation [16]. These inflammatory mediators not only recruit more immune cells but also promote macrophages and chondrocytes to produce a series of inflammatory factors as well as metalloproteinases, which can further induce synovitis and accelerate the cartilage matrix degradation, leading to a vicious cycle of joint inflammation [17].

NADPH oxidases (NOXs) are a group of membrane proteins that are widely expressed in tissues and organs [18]. These enzymes contribute to reactive oxygen species (ROS) production by modulating the intracellular electron transfer process, especially in an inflammatory environment [19, 20]. Among the NOXs family, NOX4 has the unique feature of producing hydrogen peroxide, unlike the production of superoxide by NOX1-3 [21–25]. A previous study demonstrated that the expression of NOX4 in the articular cartilage is higher in patients with OA than that in healthy individuals [26]. However, whether NOX4 plays a vital role in the pathogenesis of TMJOA and synovial macrophage-related inflammation remains unclear. GLX351322 (GLX), a novel selective NOX4 inhibitor, is a good candidate for the treatment of glucose intolerance in high-fat diets, as it prevents ROS production [27]. However, whether GLX can be used as a therapeutic agent for TMJOA remains unclear. Therefore, in this work, we explored the effect of GLX on lipopolysaccharide (LPS)-induced ROS production and inflammatory reactions in macrophages and the underlying molecular mechanisms. Furthermore, *in vivo* efficacy was explored by establishing complete Freund's adjuvant (CFA)-induced TMJ inflammation with GLX treatment. GLX was found to suppress LPS-induced ROS production and inflammation by suppressing the ROS/MAPK/NF-*κ*B signaling pathways, thus providing a new curative strategy for TMJOA.

## 2. Materials and Methods

### 2.1. Reagents and Materials

GLX351322, an inhibitor of NOX4, was obtained from Selleck (Houston, TX, USA). LPS from *Escherichia coli* O55:B5 was purchased from Sigma-Aldrich (Shanghai, China). Minimal essential medium alpha (*α*-MEM) was obtained from HyClone (Logan, UT, USA). Fetal bovine serum (FBS) was purchased from Avantor (Gaithersburg, MD, USA). TB Green Premix Ex Taq II and the Prime Script RT Reagent Kit (Perfect Real Time) were obtained from Takara Biotechnology (Otsu, Shiga, Japan). Cell Counting Kit-8 (CCK-8), ROS assay kit (S0033), and Hoechst 33342 (C1022) were purchased from Beyotime Biotechnology (Shanghai, China). Dihydroethidium (DHE) was purchased from MedChem Express (Shanghai, China). Primary antibodies against *β*-actin (CST #4970), p65 (CST #8242), phospho-p65 (CST #3033), I*κ*B*α* (CST #4814), phospho-I*κ*B*α* (CST #2859), p38 (CST #8690), phospho-p38 (CST #4511), ERK (CST # 4695), phospho-ERK (CST #4370), JNK (CST #9252), and phospho-JNK (CST #4668) were obtained from Cell Signaling Technology (CST, Danvers, MA, USA). Primary antibody against iNOS (Thermo Fisher #PA1-036) was purchased from Thermo Fisher Scientific (Waltham, MA, USA). Primary antibody against NOX4 (Affinity #DF6924) was obtained from Affinity Biosciences (Cincinnati, OH, USA).

### 2.2. mRNA Microarray Analysis

An mRNA microarray was used to investigate the differentially expressed genes (DEGs) between normal and inflammatory synovial tissues in the TMJ. Briefly, six 8-week-old male Sprague-Dawley (SD) rats were divided into two groups (*n* = 3): (1) control group and (2) CFA group. Rat TMJ inflammation was established by intra-articular injection of 5 mg/mL CFA (Chondrex, Redmond, WA, USA) in a 50 *μ*L volume. The rats were euthanized after 3 days to collect synovial tissues. Total RNA was extracted by using a TRIzol reagent, and gene expression was examined by mRNA microarray analysis (KangChen Bio-tech, Shanghai, China). SRplot (http://www.bioinformatics.com.cn/srplot) was used for Kyoto Encyclopedia of Genes and Genomes (KEGG) pathway enrichment analysis.

### 2.3. Cell Cultures

RAW 264.7 murine macrophages were cultured in *α*-MEM with 10% FBS and 100 U/mL penicillin-streptomycin in a humid environment at 37°C with 5% CO_2_. A confluence of 80–90% indicates that the cells are ready to be seeded or passed. During the process of dissociation, scrapers were used instead of trypsin to remove the attached cells from the macrophage cell line.

### 2.4. Cytotoxicity Assay

At a density of 8 10^3^ cells/well, RAW 264.7 macrophages were plated into 96-well plates for the quantitative CCK-8 assay. A variety of concentrations of GLX were applied to the cells (0, 1.25, 2.5, 5, 10, 20, and 40 *μ*M), and the cells were incubated for 24 and 48 h. At each time point, ten microliters of CCK-8 solution was added to each well. After 2 h of incubation, absorbance at 450 nm was measured by a microplate reader, with 630 nm as the reference wavelength. Quantitative results were presented as cell viability relative to the control group, with the viability of the control group set at 100%.

### 2.5. Intracellular ROS Detection

Intracellular ROS level was detected by using DCFH-DA probes (1 : 1000; S0033S; Beyotime Biotechnology, China). Briefly, RAW 264.7 macrophages were cultured for 24 h in the presence of 100 ng/mL LPS and 10 or 40 *μ*M GLX. Next, the cells were cocultured with 10 *μ*M DCFH-DA for 25 min at 37°C. Then, the cells were incubated with Hoechst 33342 for 5 min and washed three times with warm PBS. Confocal laser scanning microscopy (CLSM) was used for capturing fluorescent images. ImageJ software (National Institutes of Health) was used for the semiquantitative analysis of DCF fluorescence relative to the control.

### 2.6. Intracellular Superoxide Detection

Diahydroethidium (DHE; MedChem Express, China) was used to detect intracellular superoxide level. Briefly, RAW 264.7 macrophages were cultured with 100 ng/mL LPS and 10 or 40 *μ*M GLX for 24 h, then incubated with 5 *μ*M DHE for 30 min at 37°C. Then, the cells were incubated with Hoechst 33342 for 5 min and washed three times with warm PBS. CLSM was then used to observe the cells. ImageJ software (National Institutes of Health) was used for semiquantitative analysis of DHE fluorescence relative to the control.

### 2.7. NADPH/NADP^+^ Measurement

An NADP^+^/NADPH Assay Kit with WST-8 (S0179; Beyotime Biotechnology, China) was used to measure intracellular levels of NADP^+^ and NADPH. Using the standard curve for quantification, the supernatant was measured at 450 nm after the cells were lysed and centrifuged. The formula was as shown follows: [NADP^+^] = [NADP_total_]–[NADPH].

### 2.8. Quantitative PCR Analysis

To evaluate the expression of inflammation-related genes, RAW 264.7 macrophages were seeded in 6-well plates at a density of 5 × 10^5^ cells/well and then cultured with LPS plus 10 or 40 *μ*M GLX for 24 h. The Axygen RNA Miniprep Kit (Axygen, Union City, CA, USA) was used for total RNA extraction. An ABI 7500 Sequencing Detection System (Applied Biosystems, Foster City, CA, USA) was used to perform a quantitative PCR (qPCR) assay after reverse transcription of RNA templates, using the TB Green Premix Ex Taq II. By mixing 5 *μ*L TB Green, 3 *μ*L ddH_2_O, 1 *μ*L cDNA, 0.4 *μ*L on each primer, and 0.2 *μ*L ROX Dye2, a 10 *μ*L reaction system was established. Cycling conditions included 40 cycles of 5 s at 95°C and 30 s at 60°C. Melting curves were used to verify the specificity of the amplification. The comparative 2^−*ΔΔ*CT^ method was used to calculate the relative gene expression; *GAPDH* was used as a housekeeping gene.  Table 1 shows the list of primer sequences.

### 2.9. Western Blotting

To collect the whole cell protein, RAW 264.7 macrophages were seeded in 6-well plates, at a density of 5 × 10^5^ cells/well. GLX was added to the cells after adhesion, with the concentrations of 10 or 40 *μ*M. To extract total protein, whole cell lysis buffer with a protease inhibitor cocktail (P8340; Sigma-Aldrich) was used to lyse the cells. Then, the supernatant was collected after centrifugation at 12,000 × *g* for 15 min. Measurement of protein concentration was carried out using the bicinchoninic acid (BCA) assay (P0012; Beyotime Biotechnology, China). SDS-PAGE was used to separate the proteins collected in loading buffer, and PVDF membranes of 0.22 *μ*m were used to transfer them. Following blocking in 5% BSA in TBST (Tris-buffered saline with Tween 20) for 1 h, the membranes were incubated with the primary antibodies (*β*-actin, 1 : 1000; p65, 1 : 1000; p-p65, 1 : 1000; I*κ*B*α*, 1 : 1000; p-I*κ*B*α*, 1 : 1000; JNK, 1 : 1000; p-JNK, 1 : 1000; ERK, 1 : 1000; p-ERK, 1 : 1000; p38, 1 : 1000; and p-p38, 1 : 1000) overnight at 4°C, followed by incubation with the secondary antibodies for 1 h at room temperature and visualization of the blots using Odyssey image scanning software (LI-COR, Inc., Lincoln, NE, USA).

### 2.10. Immunofluorescence Staining

RAW 264.7 macrophages were challenged with LPS in the presence of 10 or 40 *μ*M GLX for 24 h. Primary antibodies against iNOS (1 : 100; PA1-036, Thermo Fisher Scientific, Waltham, MA) were used for incubation after the fixation, permeabilization, and blocking. A fluorescent secondary antibody was incubated for 1 hour, followed by DAPI staining. The cells were then observed via CLSM and analyzed using ImageJ software (National Institutes of Health).

### 2.11. CFA-Induced TMJ Inflammation

To evaluate the *in vivo* therapeutic efficacy of GLX, we established CFA-induced TMJ inflammation based on previous studies [28, 29]. Briefly, eighteen 8-week-old male SD rats were randomly divided into three groups: (1) saline (*n* = 6), (2) CFA (*n* = 6), and (3) GLX treated (*n* = 6). CFA (5 mg/mL, 50 *μ*L) was injected bilaterally into the TMJ cavities of rats. Rats in the GLX-treated group were injected intra-articularly with GLX (40 *μ*M, 50 *μ*L), and injections were performed every 5 days thereafter. Seven and 14 days after GLX injections, rats were euthanized, and the TMJ were dissected and fixed in 4% paraformaldehyde (PFA) for 48 h for further analysis.

### 2.12. Micro-Computed Tomography

A high-resolution micro-CT (*μ*CT-100, SCANCO Medical AG, Switzerland) with a resolution of 10 *μ*m was used for Micro-CT scanning. The X-ray energy was set at 70 kV and 200 *μ*A with a 300 ms exposure time.

### 2.13. Histological Analysis

After micro-CT scanning, the samples were decalcified in 10% EDTA (pH 7.4) for four weeks, then embedded in paraffin. Hematoxylin and eosin (H&E) and safranin O-fast green (S&F) staining were used for histological sections. Osteoarthritis Research Society International (OARSI) scores and synovitis scores were analyzed as previously reported [30, 31]. Immunofluorescence staining was performed using NOX4 primary antibody (1 : 100; DF6924; Affinity, Cincinnati, OH, USA). The histologic sections were captured under a high-quality microscope (Leica DM4000B, Wetzlar, Germany). NOX4 fluorescence relative to the saline group in synovium was quantified using ImageJ software (National Institutes of Health).

### 2.14. Statistical Analysis

GraphPad Prism 8.0 statistical software was used for all statistical analyses. All data are presented as mean ± standard deviation (SD). The differences between two groups were analyzed using unpaired Student's *t*-tests (two-tailed) after the homogeneity test of variance. One-way analyses of variance (ANOVA) with Tukey's post-hoc tests were used for multiple group comparisons. Differences were considered statistically significant at *P* < 0.05.

## 3. Results

### 3.1. Transcriptomic Evaluation of the TMJ Synovium from Normal and CFA-Injected Rats

**Figure 1:**
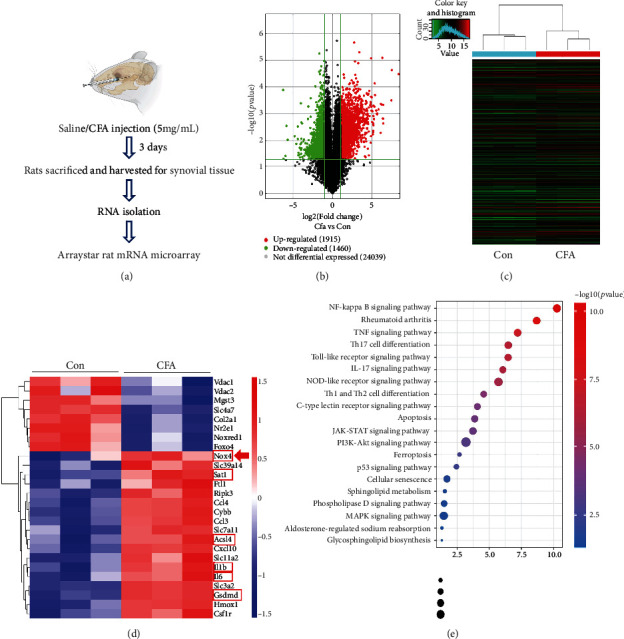
Transcriptomic analysis of the TMJ synovium samples from normal and CFA-injected rats. (a) Schematic diagram of the experimental design and procedure for sample preparation for mRNA microarray. (b) Volcano plots of differentially expressed genes (DEGs). (c) Clustered heat map of all targets. (d) Heat map of the inflammation-related genes (fold change ≥ 2.0 and *P* < 0.05). (e) KEGG pathway enrichment analysis on differentially expressed genes between the control and CFA-injected groups.

To investigate the differential gene expression between normal and inflammatory synovial tissues in the TMJ, we established TMJ inflammation in rats by injecting CFA into the articular cavity. As illustrated in Figure 1(a), synovial tissues were collected 3 days after CFA injection and were then subjected to mRNA microarray analysis. The volcano plots demonstrate that 1915 genes were markedly upregulated, with 1460 downregulated genes after intra-articular injection of CFA (Figure 1(b)). The heat map shows that the expression of several oxidative stress-related and/or inflammation-related genes was upregulated in the CFA group, such as *Nox4*, *Sat1*, *Acsl4*, *Il1b*, *Il6*, and *Gsdmd*, compared with that in the control group (Figures 1(c) and 1(d)). To further investigate the function of the differentially expressed genes, the top 20 enriched pathways were identified by KEGG analysis. Rheumatoid arthritis and the NF-*κ*B, TNF, Toll-like receptor, IL-17, and NOD-like receptor signaling pathways were the most associated with the differentially expressed genes (Figure 1(e)). Collectively, these data suggest that *Nox4* may be involved in the pathogenetic process of TMJ inflammation and could be a potential therapeutic target for in-depth investigation.

### 3.2. Effects of GLX on the Cytotoxicity of RAW 264.7 Macrophages

**Figure 2:**
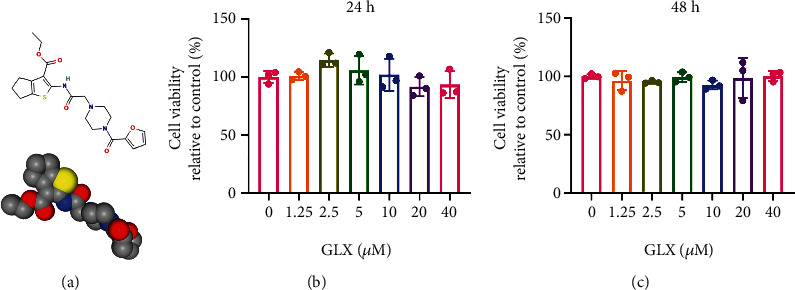
Effects of GLX on the cytotoxicity of RAW 264.7 macrophages. (a) Chemical structure of GLX, with a molecular formula of C_21_H_25_N_3_O_5_S and a molecular weight of 431.51 g/mol. (b) CCK-8 assay of RAW 264.7 macrophages with different concentrations of GLX for 24 h (*n* = 3 independent samples). (c) CCK-8 assay of RAW 264.7 macrophages with different concentrations of GLX for 48 h (*n* = 3 independent samples). The data represent the mean ± SD from three independent replicates (one-way ANOVA with Tukey's post hoc tests).

The chemical structure of GLX is shown in Figure 2(a). To obtain the safe concentration range of GLX, we first explored the cytotoxicity of GLX by using CCK-8 assay. RAW 264.7 murine macrophages were cultured with different concentrations of GLX for 24 and 48 h. As shown in Figures 2(b) and 2(c), GLX exhibited marginal cytotoxicity to RAW 264.7 macrophages even when concentration reached 40 *μ*M, indicating the satisfactory biocompatibility of GLX.

### 3.3. GLX Reduces LPS-Induced Intracellular ROS Production in RAW 264.7 Macrophages

**Figure 3:**
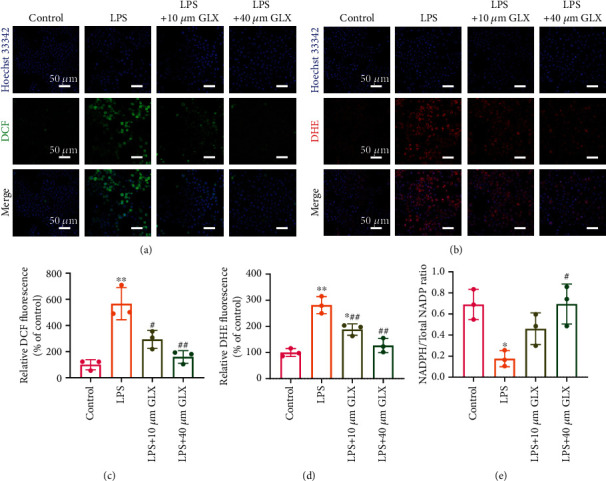
GLX reduces LPS-induced intracellular ROS production in RAW 264.7 macrophages. (a) Confocal images of RAW 264.7 macrophages after negative control, LPS, LPS with 10 *μ*M GLX treatment, and LPS with 40 *μ*M GLX treatment stained with DCFH-DA. (b) Confocal images of RAW 264.7 macrophages after negative control, LPS, LPS with 10 *μ*M GLX treatment, and LPS with 40 *μ*M GLX treatment stained with DHE. (c) Quantification of the relative DCF fluorescence (% of control) (*n* = 3 independent samples). (d) Quantification of the relative DHE fluorescence (% of control) (*n* = 3 independent samples). (e) Intracellular NADPH/Total NADPH level of RAW 264.7 macrophages after negative control, LPS, LPS with 10 *μ*M GLX treatment, and LPS with 40 *μ*M GLX treatment (*n* = 3 independent samples). The data represent the mean ± SD from three independent replicates (one-way ANOVA with Tukey's post hoc tests). ^∗^*P* < 0.05, ^∗∗^*P* < 0.01 compared with the control group; ^#^*P* < 0.05, ^##^*P* < 0.01 compared with the LPS group.

After determining the safe concentration range of GLX (≤40 *μ*M), we examined its anti-ROS capacity. RAW 264.7 macrophages were stimulated with 100 ng/mL LPS for 24 h and treated with 10 and 40 *μ*M GLX. DCFH-DA staining showed that intracellular total ROS production was significantly upregulated by LPS stimulation but markedly reduced after treatment with GLX in a dose-dependent manner (Figure 3(a)). Quantitative analysis illustrated that the relative DCF fluorescence after LPS stimulation and 40 *μ*M GLX treatment was almost the same as that of the control group (Figure 3(c)). DHE was used to evaluate the effect of GLX on superoxide anion levels. As shown in Figures 3(b) and 3(d), both 10 and 40 *μ*M GLX treatment efficiently reduced DHE fluorescence. Furthermore, the effect of GLX on intracellular NADPH, the substrate of NOX4, was examined in LPS-activated macrophages treated with GLX. As shown in Figure 3(e), LPS stimulation significantly decreased intracellular NADPH levels, indicating the overconsumption of NADPH in the inflammatory environment. However, treatment with 40 *μ*M GLX effectively restored the intracellular NADPH levels. Taken together, the results above suggest effective anti-ROS performance of GLX in a dose-dependent manner.

### 3.4. GLX Suppresses the LPS-Induced Inflammatory Reaction in RAW 264.7 Macrophages

**Figure 4:**
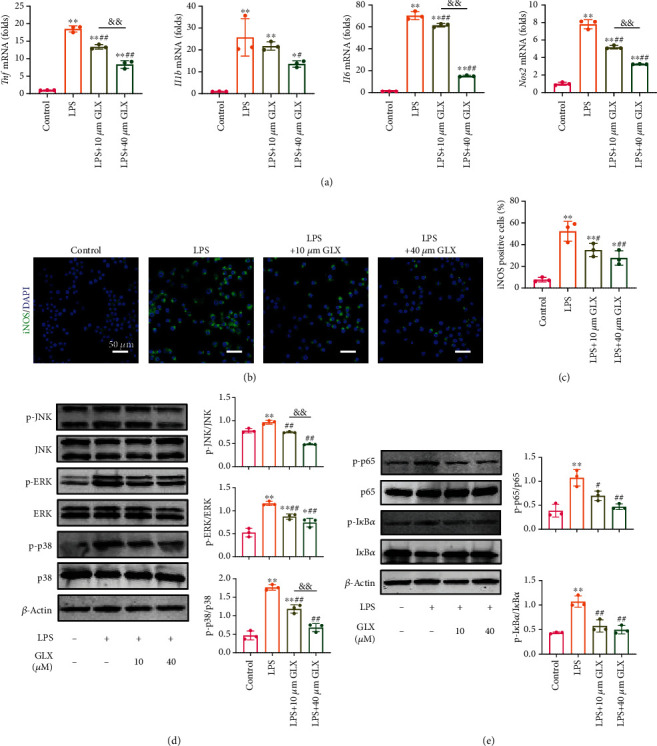
GLX suppresses the LPS-induced inflammatory reaction in RAW 264.7 macrophages. (a) Pro-inflammatory gene expression evaluated by RT-qPCR in RAW 264.7 macrophages after negative control, LPS, LPS with 10 *μ*M GLX treatment, and LPS with 40 *μ*M GLX treatment (*n* = 3 independent samples). (b) Immunofluorescence of iNOS in RAW 264.7 macrophages after negative control, LPS, LPS with 10 *μ*M GLX treatment, and LPS with 40 *μ*M GLX treatment. (c) Quantification of the iNOS positive cells (%) (*n* = 3 independent samples). (d) Western blotting of the activation of MAPKs (p38, ERK, and JNK) signaling pathways after negative control, LPS, LPS with 10 *μ*M GLX treatment, and LPS with 40 *μ*M GLX treatment (*n* = 3 independent samples). (e) Western blotting of the activation of NF-*κ*B signaling pathway after negative control, LPS, LPS with 10 *μ*M GLX treatment, and LPS with 40 *μ*M GLX treatment (*n* = 3 independent samples). The data represent the mean ± SD from three independent replicates (one-way ANOVA with Tukey's post hoc tests). ^∗^*P* < 0.05, ^∗∗^*P* < 0.01 compared with the control group; ^#^*P* < 0.05, ^##^*P* < 0.01 compared with the LPS group; ^&&^*P* < 0.01 compared with the 10 *μ*M GLX treatment group.

Encouraged by the satisfactory anti-ROS ability of GLX, we investigated the anti-inflammatory efficacy of GLX on LPS-activated macrophages. As expected, the results of qPCR demonstrated that the expression of pro-inflammatory genes (*Tnf*, *Il1b*, *Il6*, and *Nos2*) increased by LPS stimulation but markedly reduced after treatment with 10 and 40 *μ*M GLX dose-dependently (Figure 4(a)). Of note, 40 *μ*M GLX treatment exhibited more inhibitory effect on the gene expression of *Tnf*, *Il6*, and *Nos2*, compared with that of the 10 *μ*M GLX treatment group. Immunofluorescence for iNOS also illustrated that iNOS expression was efficiently downregulated by 10 and 40 *μ*M GLX treatment (Figures 4(b) and 4(c)). To gain further insights into how GLX inhibits the inflammatory process, we explored the potential mechanism by western blotting. Several important signaling pathways influenced by ROS have been studied, including the MAPK and NF-*κ*B signaling pathways. As shown in Figure 4(d), the phosphorylation of p38, ERK, and JNK was activated upon stimulation with LPS, whereas it was greatly suppressed after treatment with GLX. Of note, 40 *μ*M GLX treatment exhibited more inhibitory effect on the phosphorylation of JNK and p38, compared with that of the 10 *μ*M GLX treatment group. Similarly, LPS stimulation activated the phosphorylation of p65 and I*κ*B*α*. However, when treated with GLX, the activation was markedly reduced (Figure 4(e)). Taken together, these results demonstrate that GLX suppresses the LPS-induced inflammatory reaction in RAW 264.7 macrophages via inhibiting the activation of MAPK and NF-*κ*B signaling pathways. Meanwhile, 40 *μ*M GLX suppresses the LPS-induced macrophage inflammatory reaction more efficiently, and this concentration would be used in the subsequent *in vivo* application.

### 3.5. GLX Alleviates CFA-Induced TMJ Inflammation in Rats

**Figure 5:**
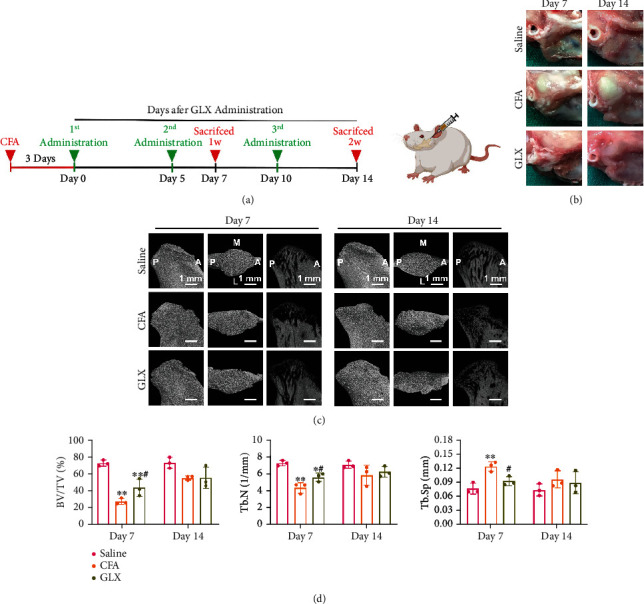
General histopathologic changes and radiological evaluation of GLX on CFA-induced TMJ inflammation in rats. (a) Schematic of the *in vivo* experiments. (b) Histopathologic changes in the rat TMJ after treatment on days 7 and 14 for each group. (c) Micro-CT scanning of rat TMJ after treatment on days 7 and 14 for each group. (d) Quantification of micro-CT scanning of rat TMJ after treatment on days 7 and 14 for each group (*n* = 3 independent animals). The data represent the mean ± SD from three independent replicates (one-way ANOVA with Tukey's post hoc tests). ^∗^*P* < 0.05, ^∗∗^*P* < 0.01 compared with the saline group; ^#^*P* < 0.05, ^##^*P* < 0.01 compared with the CFA group.

After figuring out the satisfactory biosafety, anti-ROS, and anti-inflammatory efficacy of GLX *in vitro*, we further explored the *in vivo* therapeutic efficacy by using CFA-induced TMJ inflammation in the rats. The design and procedure of *in vivo* experiment is shown in Figure 5(a). Accurate intra-articular injection was performed by using anterior superior puncture technique in rats. Three days after injection with CFA, GLX was administered to the inflammatory TMJ every 5 days. The rat TMJ samples were collected at 7 and 14 days after the GLX treatment to evaluate the *in vivo* anti-inflammatory effects of GLX. To observe therapeutic efficacy, the inflammatory TMJ were collected and photographed. Pathomorphological images showed that the injection of CFA induced joint inflammation (Figure 5(b)). In contrast, treatment with GLX efficiently attenuated TMJ inflammation, with reduced swelling in synovial tissue at each time point. Micro-CT scanning was used to evaluate the radiographic outcomes of GLX. As shown in Figure 5(c), compared with the saline group, progressive arthritis was established on day 7. Extensive damage in the condylar surface as well as significant reduction in the bone mass of the subchondral bone was observed. On day 14, extensive condylar remodeling and increased density of the subchondral bone were observed, indicating an advanced stage of TMJ inflammation. These data indicate that TMJOA was successfully established by the intra-articular injection of CFA. After GLX administration, both the condylar surface integrity and subchondral bone structure were efficiently protected, with significant improvements in bone volume/tissue volume (BV/TV), trabecular number (Tb.N), and trabecular separation (Tb.Sp) on day 7 (Figure 5(d)). On day 14, condylar remodeling was efficiently attenuated in GLX-treated rats, with a relatively intact articular surface. However, no significant differences in BV/TV, Tb.N, and Tb.Sp were detected between the CFA and GLX-treated groups (Figure 5(d)). Anyway, the results above illustrated that GLX can alleviate progressive TMJ inflammation and protect subchondral bone structure in a highly efficient manner.

**Figure 6:**
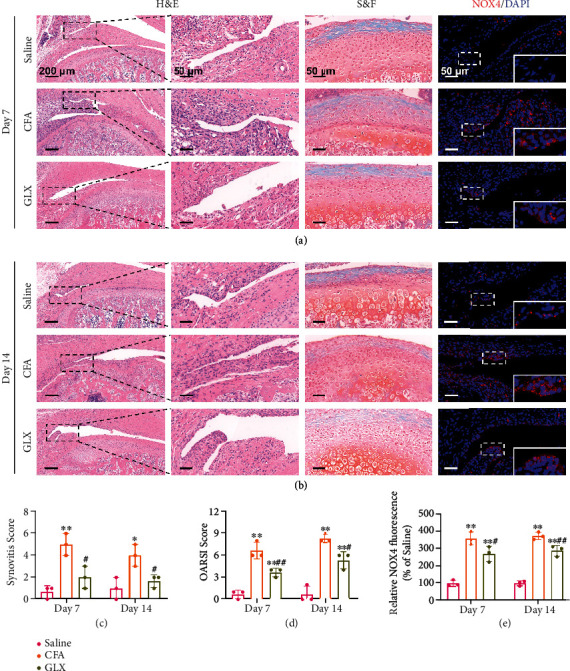
Histological analysis of the therapeutic effect of GLX on CFA-induced TMJ inflammation in rats. (a) Representative images of H&E, S&F, and immunofluorescence staining of NOX4 on day 7 after treatment from each group. (b) Representative images of H&E, S&F, and immunofluorescence staining of NOX4 on day 14 after treatment from each group. (c) Quantification of the synovitis score (*n* = 3 independent animals). (d) Quantification of the OARSI score (*n* = 3 independent animals). (e) Quantitative analysis of the relative NOX4 fluorescence (% of saline group) (*n* = 3 independent animals). The data represent the mean ± SD from three independent replicates (one-way ANOVA with Tukey's post hoc tests). ^∗^*P* < 0.05, ^∗∗^*P* < 0.01 compared with the saline group; ^#^*P* < 0.05, ^##^*P* < 0.01 compared with the CFA group.

Consistent with the micro-CT scanning, the histological analysis illustrated that the model of TMJ arthritis was well established by local CFA injection. The H&E staining showed the inflamed synovial tissue and broken condylar cartilage on day 7 (Figure 6(a)). On day 14, advanced TMJ inflammation could be detected, with a hyperplastic synovium and a damaged condylar surface (Figure 6(b)). In contrast, treatment with GLX efficiently attenuated the inflammatory progression, with reduced synovitis and OARSI scores (Figures 6(c) and 6(d)). These results indicated that GLX effectively protected the condylar cartilage structure owing to its inhibitory effect on synovial inflammation. Moreover, NOX4 expression in TMJ inflammation was evaluated by histological immunofluorescence. The semiquantitative results demonstrated that the NOX4 expression was markedly upregulated in the inflamed synovial tissue, and the NOX4-positive cells accumulated in the synovial lining layers. The relative fluorescence of NOX4 was remarkably reduced by GLX treatment in the inflammatory synovium, which represented the reduced expression of NOX4 (Figure 6(e)). Taken together, the *in vivo* data indicated that GLX significantly alleviated the synovial inflammation in the TMJ, thus protecting the condylar structure effectively.

## 4. Discussion

TMJOA is one of the most common degenerative diseases of the TMJ. Although patients with end-stage TMJOA are advised to undergo total joint replacement, the high cost of TMJ prostheses severely restricts their use in most developing countries, which has caused a huge social and economic burden [32, 33]. Abnormal loading, unhealthy biting habits, unilateral mastication, clenching, trauma, and psychological factors have been corroborated to contribute to the progression of TMJOA. Although articular cartilage degeneration, subchondral bone remodeling, and synovial inflammation are considered the main pathological characteristics of TMJOA, the underlying molecular mechanisms and therapeutic targets remain to be explored.

As the main source of inflammatory enzymes and cytokines, synovial macrophages play a vital role in the development of TMJOA [34]. In recent years, oxidative stress has been shown to participate in and accelerate OA progression [35]. NOX family members act as key enzymes in ROS production, and NOX4 exerts a unique effect on immune cells. A series of studies have indicated the important role of NOX4 in OA, which may promote the development of OA by activating ROS-related signaling pathways such as MAPK, NF-*κ*B, PI3K-AKT, and NLRP3 [36–38]. However, the above studies were mainly involved in chondrocyte differentiation and apoptosis, while lacking information on the effect of NOX4 on inflammatory synovial macrophages. Stimulated by inflammatory cytokines, synovial macrophages may exhibit respiratory burst, with a rapid increase in mitochondrial oxygen consumption and the production of ROS, thus leading to oxidative stress. Oxidative stress further promotes the production of inflammatory cytokines, and triggers a vicious cycle [39]. Therefore, therapies targeting excessive ROS production, as well as NOX4 in inflammatory macrophages, have great potential for investigation. Several NOX4 inhibitors have been reported to exert anti-inflammatory effects on inflammatory macrophages, including setanaxib (GKT137831) [40], apocynin (NSC 2146) [41], and 2-acetylphenothiazine (ML171) [42]. GLX, an emerging NOX4 inhibitor, inhibits ROS production and NLRP3 inflammasome activation in chondrocytes [37]. However, no reports have discussed the role of GLX in ROS production and inflammatory responses in inflammatory macrophages *in vitro* and *in vivo*.

In the current study, we first demonstrated the important role of NOX4 in the pathogenesis of TMJ inflammation in rat TMJ synovial tissues using mRNA microarray analysis. The CFA group exhibited higher expression of NOX4 and other inflammatory and ROS-related genes, indicating the potential role of NOX4 in TMJOA. Based on these results, we explored the effect of GLX on LPS-induced macrophage inflammation. ROS production was dose-dependently inhibited with GLX treatment. Notably, although GLX was reported to be a highly selective inhibitor of NOX4, a hydrogen peroxide-generating oxygen sensor, the intracellular superoxide anion level was also partly reduced by GLX treatment, which could be explained by the moderate suppression of NOX2. Meanwhile, the intracellular NADPH level was almost restored to normal with 40 *μ*M GLX treatment. Encouraged by the satisfactory anti-ROS efficacy of GLX, we investigated anti-inflammatory phenotypes and molecular mechanisms in LPS-challenged macrophages. At the transcriptional level, GLX treatment significantly reduced the expression of pro-inflammatory genes, including *Tnf*, *Il1b*, *Il6*, and *Nos2*. The immunofluorescence expression of iNOS, a well-known pro-inflammatory cytokine, was blunted by GLX treatment. The phosphorylation of proteins in the MAPK (p38, ERK, and JNK) and NF-*κ*B (p65 and I*κ*B*α*) pathways has been widely reported for ROS-induced inflammatory reactions. Therefore, we examined the effects of GLX on these crucial inflammation-related pathways. As expected, GLX efficiently suppressed the activation of p38, ERK, JNK, p65, and I*κ*B*α*, indicating that GLX may alleviate the inflammatory response by inhibiting the ROS/MAPK/NF-*κ*B signaling pathways.

Consistent with the *in vitro* anti-ROS and anti-inflammatory properties, the *in vivo* results further confirmed the therapeutic effect of GLX on CFA-induced TMJ inflammation. The TMJ inflammation model induced by CFA is a widely acknowledged and reproducible experimental method for investigating TMJOA [29, 43]. In this study, we used CFA-induced TMJ inflammation with GLX treatment via intra-articular injections. Both micro-CT and histological staining demonstrated attenuated joint inflammation in the GLX-treated group, with decreased synovitis and OARSI scores. Furthermore, immunofluorescence staining showed that GLX treatment significantly reduced NOX4 expression in synovial tissues, indicating the therapeutic effect of GLX on TMJ inflammation via downregulation of NOX4 expression.

However, as a preliminary study, this study has several limitations. First, although this study indicates the anti-inflammatory effect of GLX on inflammatory macrophages by inhibiting the ROS/MAPK/NF-*κ*B signaling pathways, whether GLX influences other ROS-related pathways is yet to be clarified. Moreover, whether GLX affects chondrogenic and osteoclast differentiation should be further investigated *in vitro* and *in vivo*.

## 5. Conclusions

**Figure 7:**
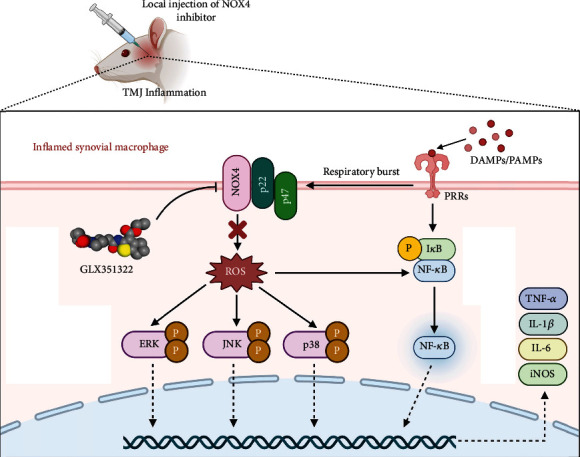
Schematic illustration of GLX for therapy in TMJ inflammation (created with http://BioRender.com).

In summary, this study demonstrates the potential therapeutic effect of GLX on TMJOA and indicates the mechanisms of both its anti-ROS and anti-inflammatory effects, providing a novel direction for researchers to focus on the influence of oxidative stress and inflammatory responses in the pathogenesis of TMJOA (Figure 7). Both in vitro and in vivo results suggest that GLX intervention or NOX4 inhibition is a promising potential curative strategy for TMJOA and other inflammatory diseases in the future.

## Figures and Tables

**Table 1:** Primer sequences for qPCR.

Gene	Forward	Reverse
*Gapdh*	ACCCAGAAGACTGTGGATGG	CACATTGGGGGTAGGAACAC
*Tnf*	GCCTCTTCTCATTCCTGCTTGTGG	GTGGTTTGTGAGTGTGAGGGTCTG
*Il1b*	TCGCAGCAGCACATCAACAAGAG	AGGTCCACGGGAAAGACACAGG
*Il6*	CTTCTTGGGACTGATGCTGGTGAC	AGGTCTGTTGGGAGTGGTATCCTC
*Nos2*	ACTCAGCCAAGCCCTCACCTAC	TCCAATCTCTGCCTATCCGTCTCG

## Data Availability

The data used and/or analyzed to support the findings in this study are available from the corresponding authors upon request.
